# Development and validation of a medication non-adherence scale for Malaysian hypertensive patients: a mixed-methods study

**DOI:** 10.1265/ehpm.23-00223

**Published:** 2023-12-05

**Authors:** Sheng-Qian Yew, Kit-Aun Tan, Ahmad Iqmer Nashriq Mohd Nazan, Rosliza Abdul Manaf

**Affiliations:** 1 Department of Public Health Medicine, Faculty of Medicine, Universiti Kebangsaan Malaysia, Jalan Yaacob Latif, Bandar Tun Razak, 56000 Cheras, Kuala Lumpur, Malaysia; 2 Department of Psychiatry, Faculty of Medicine and Health Sciences, Universiti Putra Malaysia, 43400 UPM Serdang, Selangor, Malaysia; 3 Department of Community Health, Faculty of Medicine and Health Sciences, Universiti Putra Malaysia, 43400 UPM Serdang, Selangor, Malaysia

**Keywords:** Hypertension, Medication adherence, Scale development, Scale validation, Psychometric properties

## Abstract

**Background:**

Non-adherence to anti-hypertensive medications can lead to hypertension-related complications. One of the most effective preventive measures to mitigate these complications is to understand the underlying determinants of medication non-adherence using various scales. Unfortunately, existing scales for measuring non-adherence to anti-hypertensive medications have certain limitations, such as insufficient consideration of validity, dimensionality, and cultural adaptation. In response, the current study aimed to develop and validate a measure of non-adherence to anti-hypertensive medications—known as the Malaysian Anti-hypertensive Agent Non-Adherence Scale (MAANS)—for use in local hypertensive patients.

**Methods:**

A two-phase mixed-methods approach was used. Phase 1 involved qualitative interviews with hypertensive patients from two health clinics in Kuala Lumpur, Malaysia. The themes extracted from these interviews were used to generate items for the MAANS. In Phase 2, data from 213 participants were analysed using exploratory factor analysis (EFA) to establish the scale’s factor structure, thereby created the modified version of the MAANS. Confirmatory factor analysis (CFA) was then conducted on a separate dataset of 205 participants to confirm the factor structure, resulted in the final version of the MAANS. The reliability of the final MAANS version was assessed using Cronbach’s alpha coefficient. The MAANS scores were used to predict subscales of the Malay version of the WHO Quality-of-Life (QOL) BREF, demonstrating the scale’s predictive validity.

**Results:**

Ten qualitative interviews yielded 73 items. The EFA produced a modified MAANS with 21 items grouped into five factors. However, the CFA retained three factors in the final scale: Perceived Non-Susceptibility, Poor Doctor-Patient Relationship, and Unhealthy Lifestyle. The final 14-item, 3-factor MAANS demonstrated moderate reliability (Cronbach’s alpha coefficient = 0.64) and exhibited partial predictive validity, with the Poor Doctor-Patient Relationship and Unhealthy Lifestyle subscales significantly predicting Social QOL and Environmental QOL.

**Conclusion:**

The MAANS is a reliable, valid, and multidimensional scale specifically developed to evaluate non-adherence to anti-hypertensive medications in local clinical settings with the potential to further the advancement of research and practice in sociomedical and preventive medicine.

## 1. Background

Non-adherence to anti-hypertensive medications is an alarming issue worldwide with a global prevalence ranged 27.0–40.0% [1]. In Malaysia, the prevalence of non-adherence to anti-hypertensive medications was also high, ranged 24.2–51.3% [2, 3]. This not only has substantial economic implications to the country [4] but also carries the risk of uncontrolled blood pressure among the patients [5], ultimately leading to hypertension-related complications, such as cardiovascular diseases [6, 7]. As such, one of the most effective strategies for preventing these complications is to target the root cause, which is the issue of medication non-adherence [8]. To achieve a satisfactory level of adherence to medication, it is essential for both healthcare providers and patients to gain a comprehensive understanding of the underlying social determinants for medication non-adherence [9]. This understanding is a critical prerequisite for developing effective preventions to address the issue of medication non-adherence.

In the past decades, researchers have developed multiple scales to assess medical non-adherence among hypertensive patients [10]. Notable examples of these scales include the Brief Medication Questionnaire (BMQ) [11], the Morisky Medication Adherence Scale (MMAS-4) [12], the Morisky Medication Adherence Scale (MMAS-8) [13], the Maastricht Utrecht Adherence in Hypertension (MUAH-25) [14], the Maastricht Utrecht Adherence in Hypertension (MUAH-16) [15], the Treatment Adherence Questionnaire for Patients with Hypertension (TAQPH) [16], the Hill-Bone Compliance Scale (HBSC) [17], and the Self-Efficacy for Appropriate Medication Use Scale (SEAMS) [18]. While these adherence scales are widely used in different countries, they possess certain limitations, especially when employed in the specific context of hypertensive patients locally. Firstly, it should be noted that these scales were initially created for diverse populations, and some were not explicitly designed with hypertensive patients in mind [12, 13]. Consequently, it remains uncertain to what extent these scales accurately capture the adherence behaviours within the local hypertensive community [19]. Secondly, existing literature indicates that non-adherence to medication-taking is a multidimensional construct [20]. However, some of the currently employed scales, such as the MMAS-4, MMAS-8, and HBSC, are unidimensional in nature. These scales categorise patients solely as adherent or non-adherent, offering limited understanding of the underlying causes of non-adherence. Thirdly, when considering the reliability of these scales, none of the aforementioned scales demonstrate adequate internal consistency, particularly when applied to the local hypertensive community [21]. Fourthly, many of the currently used scales lack sufficient reporting of content validity, criterion validity, and/or construct validity during their development process, casting doubt on the validity of these scales. Lastly, the existing hypertension-related scales generally lack the incorporation of theoretical frameworks in their development, hindering researchers and practitioners from ascertaining whether the constructs in these adherence scales comprehensively describe a person’s adherence behaviour.

Recognising these gaps in both the literature and clinical practice, the objectives of the present study were threefold. Firstly, the study aimed to investigate the themes of non-adherence to anti-hypertensive medications among hypertensive patients receiving treatment at health clinics in Kuala Lumpur. Secondly, the study sought to develop a scale called the Malaysia Anti-hypertensive Agent Non-Adherence Scale (MAANS) based on the identified themes. Finally, the study aimed to assess the psychometric properties of the MAANS, including its factor structure, reliability, and predictive validity.

## 2. Methods

The study was conducted in two distinct phases, referred to as Phase 1 (i.e., a qualitative study to generate items for the MAANS) and Phase 2 (i.e., a quantitative study to validate the MAANS). The study protocol was published elsewhere [22]. Ethical approval for the present study was granted by the Medical Research Ethics Committee (MREC), Ministry of Health Malaysia (NMRR-19-2152-49365). Table 1 shows the scale development process of the MAANS.

**Table 1:** The scale development process of the MAANS

**Phases**	**Details**	**Outcomes**
1	Ten in-depth interviews were conducted. Thematic analysis was performed.	Five themes were obtained.
2	Item pool was created based on the five themes.	Seventy-three items were created and later translated into Malay.
	Content validity test was performed.	The preliminary version of the 67-item MAANS was obtained.
	Sixteen think-aloud sessions were conducted.	The pilot version of the 67-item MAANS was obtained.
	The calibration sample included 213 participants. Exploratory factor analysis was performed.	The modified version of the 5-factor model, 21-item MAANS was obtained.
	The validation sample included 205 participants. Confirmatory factor analysis was performed. Reliability test was performed. Predictive validity test was performed.	The final version of the 3-factor model, 14-item MAANS was obtained.

### 2.1 Phase 1

In Phase 1 of the study, hypertensive patients who were attending follow-up appointments at two health clinics in Kuala Lumpur, Malaysia between September 2019 and September 2020 were purposively sampled. To be eligible for participation in the study, patients had to meet the following criteria: (a) be 18 years of age or older, (b) have a diagnosis of hypertension (defined as consistently elevated systolic blood pressure (SBP) ≥ 140 mmHg and/or diastolic blood pressure (DBP) ≥ 90 mmHg), (c) have been receiving anti-hypertensive medications for a minimum of six months, and (d) be able to read and communicate in either English or Malay language.

However, patients were excluded from the study if they met any of the following criteria: (a) diagnosed with secondary hypertension, (b) currently undergoing active cancer treatment, (c) experiencing severe mental health disorders or cognitive impairment at the time of recruitment, or (d) pregnant.

In-depth interviews were conducted using an interview schedule (in both English and Malay languages) from October 2020 to January 2021, until thematic saturation was achieved. Following each interview, the audio recordings were transcribed verbatim using NVIVO software. Interviews conducted in English language were transcribed directly by the interviewer, while interviews conducted in Malay language were translated and transcribed into English language by the interviewer. Thematic analysis was conducted using a deductive coding approach, guided by the WHO Adherence Framework.

### 2.2 Phase 2

#### 2.2.1 Initial item pool

In Phase 2, the themes of non-adherence to anti-hypertensive medications identified during Phase 1 of the study were utilised to create an initial item pool in the English language. Subsequently, a language expert translated the initial item pool into Malay language, as the intention was to construct the MAANS in Malay language for subsequent analyses.

#### 2.2.2 Content validity

The initial item pool underwent a content validity test through an expert review. Three experts evaluated the representativeness and clarity of each item and subscale using several content validity indices, including the item-level content validity index for representativeness (I-CVI representativeness), item-level content validity index for clarity (I-CVI clarity), subscale-level content validity index for representativeness (S-CVI representativeness), and subscale-level content validity index for clarity (S-CVI clarity). The content validity indices and qualitative feedback provided by the experts was carefully reviewed and revisions were implemented. This created the preliminary version of the MAANS.

#### 2.2.3 The preliminary version of the MAANS

In the preliminary version of the MAANS, a four-point Likert response scale ranging from 1 (*strongly disagree*) to 4 (*strongly agree*) was used for each item. For scoring purposes, higher total scores on the scale indicate a higher degree of non-adherence to anti-hypertensive medications.

#### 2.2.4 The pilot version of the MAANS

Sixteen hypertensive patients were randomly selected to participate in the pilot study. These patients met the same inclusion criteria as mentioned earlier. During the pilot study, they were instructed to “think-aloud” and provide verbal feedback when they encountered any words or statements that were difficult to interpret. They were also asked to revisit any problematic items and offer suggestions for improving them. After the pilot study, the pilot version of the MAANS was finalised based on the feedback and input received from the participating patients.

#### 2.2.5 The modified version of the MAANS

To determine the factor structure of the pilot version of the MAANS, an exploratory factor analysis (EFA) was conducted. A total of 213 hypertensive patients, who adhere to the same inclusion and exclusion criteria mentioned earlier, were randomly sampled from both health clinics. Upon agreement, they were invited to complete the pilot version of the MAANS.

The EFA stage was carried out from March 2021 to April 2021. The sociodemographic characteristics of the participants were collected, along with their scores for each item in the pilot version of the MAANS. The suitability of the data for conducting an EFA was assessed using the Kaiser-Meyer-Olkin (KMO) Measure of Sampling Adequacy and Bartlett’s Test of Sphericity. For the KMO index, a value of at least 0.60 was considered appropriate for the EFA [23]. Additionally, the Bartlett’s Test of Sphericity needed to yield a significant result (*p* < 0.05) to confirm the data’s suitability for the EFA [23]. Once the data’s suitability for the EFA was confirmed, the subsequent steps involved factor rotation and extraction. Several methods were utilised to determine the optimal number of factors to retain in the model. Firstly, the Kaiser-Guttman criterion was applied, whereby factors with eigenvalues above 1 were retained [24]. Secondly, the degree of over-determination was considered, and factors consisting of at least three items were retained [25–27]. Thirdly, scree plots were examined, with the component number at the plot’s breakpoint indicating the number of factors to be retained [28, 29]. Fourthly, items with communality values below 0.20 were removed based on communalities. Lastly, items with factor loadings below 0.40 and cross-loadings on two or more factors with loading values above 0.32 were eliminated based on the size of loading [25, 30]. To ensure the robustness of the factor structure, parallel analysis was conducted using the SPSS syntax by O’Connor [31]. The EFA was performed using Statistical Package for the Social Sciences (SPSS) version 26. Following the completion of the EFA, the modified version of the MAANS was obtained.

#### 2.2.6 The final version of the MAANS

To validate the factor structure of the modified version of the MAANS, a confirmatory factor analysis (CFA) was conducted. A sample of 205 hypertensive patients were randomly selected, employing the same inclusion and exclusion criteria as previously described. The CFA stage was conducted between April 2021 and May 2021. Upon agreement, they were invited to complete the modified version of the MAANS. The sociodemographic characteristics of the participants and their scores on all items in the modified version of the MAANS were collected. Additionally, to assess the predictive validity of the scale, an additional section consisting of 26 items from the Malay version of the World Health Organization Quality of Life (QOL) BREF was included in the research questionnaire. In the CFA, several goodness-of-fit indices were employed to evaluate the overall model fit without specific numbering. These indices include the chi-square statistic (χ^2^), relative chi-square (χ^2^/*df*), root mean square error of approximation (RMSEA), standardised root mean square residual (SRMR), and comparative fit index (CFI) [32]. The chi-square statistic, χ^2^, is considered a good fit if it is non-significant (*p* > 0.05). The relative chi-square (χ^2^/*df*) value should be less than 2 to indicate a good model fit [33, 34]. The RMSEA value should be 0.06 or lower, while the SRMR value should be less than 0.08 for a good model fit [35]. The comparative fit index (CFI) value should be greater than or equal to 0.95 [35], although a value between 0.90 and 0.95 is considered acceptable [36]. The CFA was conducted using AMOS 24 software [37]. Following the CFA, the final version of the MAANS was obtained.

#### 2.2.7 The reliability and validity of the final version of the MAANS

The internal consistency of the final version of the MAANS was assessed to determine its reliability. Cronbach’s alpha coefficient was used for this analysis, where values below 0.50 were considered insufficient, 0.50–0.69 were moderate, 0.70–0.79 were satisfactory, and ≥0.80 were considered good [38]. To evaluate the predictive validity of the final version of the MAANS, multiple regression analyses were conducted, regressing the WHO QOL BREF-Malay version on the MAANS final version. The WHO QOL BREF-Malay version comprises four subscales: physical QOL, psychosocial QOL, social QOL, and environmental QOL. The hypothesis was that the final version of the MAANS would be able to predict the QOL in these four subscales. Specifically, it was expected that poorer adherence to anti-hypertensive medication would be associated with lower QOL scores in the physical, psychosocial, social, and environmental subscales.

## 3. Results

### 3.1 Phase 1

Ten in-depth interviews were conducted and revealed that non-adherence to anti-hypertensive medications was associated the patient-related, condition-related, therapy-related, socioeconomic, and healthcare system-related themes. These five themes corresponded to the five constructs outlined in the WHO Medication Adherence Framework. Details of the qualitative findings were published elsewhere [39].

### 3.2 Phase 2

#### 3.2.1 Initial item pool

In Phase 2, themes from Phase 1 were operationalised into 73 items. These items were originally in the English language and were later translated into Malay language by a language expert.

#### 3.2.2 Content validity

Three experts were given response sheets containing 73 items in Malay language. After conducting the expert review, 7 items were removed due to inadequate item representativeness. Additionally, one double-barrelled item was divided into two separate items. No new items were introduced to the scale. Consequently, a preliminary version of the MAANS consisting of 67 items was obtained.

#### 3.2.3 The pilot version of the MAANS

Following the 16 think-aloud sessions, all participants found the instructions clear to address various aspects of non-adherence to anti-hypertensive medications. However, feedback from 14 participants indicated that the scale was too lengthy and time-consuming to complete in a clinic setting. Notably, three items caused confusion among the patients and were subsequently revised. No items were removed from the scale after the pilot study. As a result, a pilot version of the MAANS consisting of 67 items was obtained.

#### 3.2.4 The modified version of the MAANS

The EFA (calibration sample) included a total of 213 participants. Table 2 displays the sociodemographic characteristics of the participants in the EFA.

**Table 2:** Sociodemographic characteristics of participants in the EFA

**Baseline Characteristics**	***n***	**%**
**Gender**		
Male	89	41.8
Female	124	58.2
**Age group**		
18–29	8	3.8
30–49	61	28.6
50–64	89	41.8
65 and above	55	25.9
**Ethnicities**		
Malay	129	60.6
Chinese	72	33.8
Indians	5	2.3
Others	7	3.3
**Religions**		
Islam	131	61.5
Christian	37	17.4
Buddhism	39	18.3
Hinduism	5	2.3
Others	1	0.5
**Marriage Status**		
Married	198	93.0
Not Married	15	7.0
**Working Status**		
Working	98	46.0
Not working	115	54.0
**Education Level**		
Primary school	43	20.2
Secondary school	56	26.3
Certificate	22	10.3
Diploma	22	10.3
Bachelor degree	59	27.7
Master’s degree	9	4.2
Doctoral degree	2	0.9

The KMO value for the 67 items was 0.70 and the Bartlett’s test of sphericity yielded a significant result (χ^2^ = 4636.83, *p* < 0.001), indicated the suitability of the data for factor analysis. As the assumption of normality was not violated in the EFA data, a promax (oblique) factor rotation and Maximum Likelihood extraction method were employed.

The results of the EFA indicated that a 24-factor solution, as suggested by the Kaiser-Guttman criterion, explained 52.80% of the total variance among all items. This 24-factor structure was also supported by the Catell’s scree plot. However, after applying additional criteria such as size of loading, degree of over-determination, and communalities, a total of 46 items were deleted. Among these, 20 items had factor loadings below 0.40, and 26 items loaded on factors consisting of less than three items. No items were removed based on communalities < 0.20 and cross-loadings > 0.32. As a result of the item deletions, a 5-factor solution with 21 remaining items emerged. Parallel analysis, conducted using the SPSS syntax developed by O’Connor [40], also supported the retention of five factors. Therefore, the 5-factor solution with 21 items was deemed the most parsimonious and conceptually relevant. The factors were named Perceived Non-Susceptibility, Poor Doctor-Patient Relationship, Unhealthy Lifestyle, Perceived Barriers, and Limitations of Healthcare Facilities. This constituted the modified version of the MAANS.

#### 3.2.5 The final version of the MAANS

The CFA (validation sample) included a total of 205 participants. Table 3 displays the sociodemographic characteristics of the participants in the CFA.

**Table 3:** Sociodemographic characteristics of participants in the CFA

**Baseline Characteristics**	***n***	**%**
**Gender**		
Male	86	42.0
Female	119	58.0
**Age group**		
18–29	2	1.0
30–49	55	26.8
50–64	72	35.1
65 and above	76	37.1
**Ethnicities**		
Malay	146	71.2
Chinese	51	24.9
Indians	3	1.5
Others	5	2.4
**Religions**		
Islam	147	71.7
Christian	20	9.8
Buddhism	30	14.6
Hinduism	3	1.5
Others	5	2.4
**Marriage Status**		
Married	191	93.2
Not Married	14	6.8
**Working Status**		
Working	87	42.4
Not working	118	57.6
**Education Level**		
Primary school	65	31.7
Secondary school	66	32.2
Certificate	20	9.8
Diploma	20	9.8
Bachelor’s degree	31	15.1
Master’s degree	2	1.0
Doctoral degree	1	0.5

**Fig. 1:**
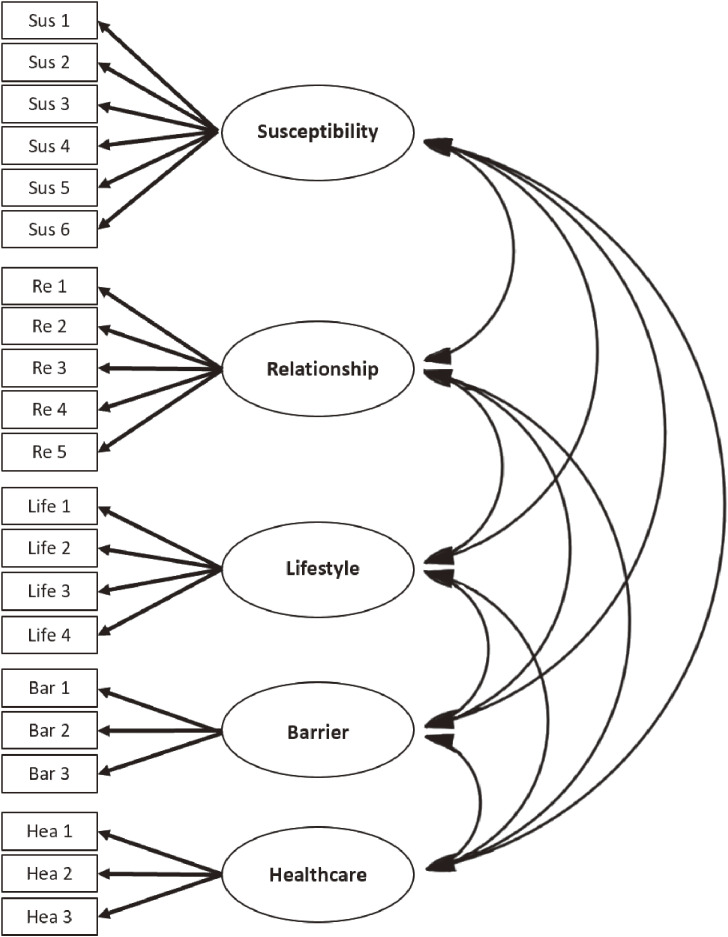
Path diagram of the hypothesised 5-factor model

In CFA, the hypothesized 5-factor model demonstrated a moderate overall fit. The chi-square statistic, χ^2^ (179, *N* = 205) = 285.89, *p* < 0.001, was found to be significant. The χ^2^/*df* of 1.49, RMSEA of 0.05, and SRMR of 0.07 suggested a good fit of the 5-factor model. However, the CFI value of 0.78 was below the cut-off point, indicating that the model did not have a good fit according to that particular index. A closer inspection of the hypothesized 5-factor model (see Fig. 1) revealed that the factor loadings of all items were statistically significant (*p*s < 0.05), except for Life1, Hea2, Bar1, and Bar2 (*p*s > 0.05). In order to improve the model-fit of the 5-factor model, these four items were removed. Consequently, the Perceived Barriers and Limitations of Healthcare Facilities factors, which consisted of less than three items after the removal, were also eliminated. This decision aligns with the degree of over-determination criterion, which stipulates that each factor must have at least three items.

**Fig. 2:**
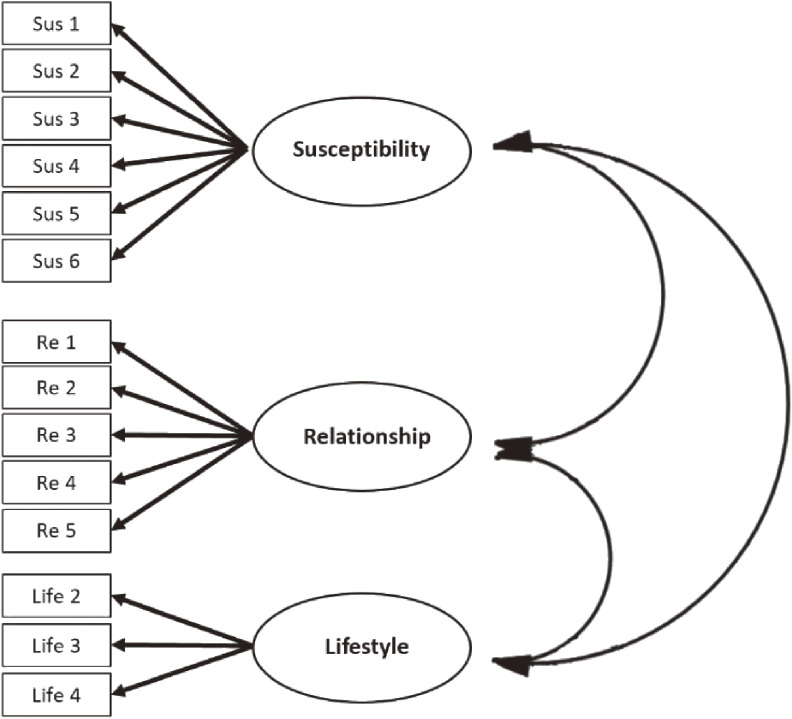
Path diagram of the hypothesised 3-factor model

Another CFA was conducted on the 3-factor model (see Fig. 2), and the fit indices for this model were as follows: χ^2^ (113, *N* = 205) = 89.22, *p* = 0.11; χ^2^/*df* = 1.21; RMSEA = 0.03; SRMR = 0.06; CFI = 0.94. These fit indices indicated an overall good model fit for the 3-factor model. Additionally, all factor loadings were statistically significant and loaded onto their respective factors (*p*s < 0.05).

After conducting the second CFA, the final version of the MAANS was determined to be best characterized as a 3-factor model consisting of Perceived Non-Susceptibility, Poor Doctor-Patient Relationship, and Unhealthy Lifestyle factors. This final version comprised a total of 14 items.

#### 3.2.6 Reliability of the final version of the MAANS

The reliability of the final version of the MAANS was assessed using internal consistency, specifically Cronbach’s alpha. The Cronbach’s alpha for the final version of the MAANS total scale was found to be 0.64. The Cronbach’s alpha estimates for the Perceived Non-Susceptibility, Poor Doctor-Patient Relationship, and Unhealthy Lifestyle subscales were 0.54, 0.64, and 0.41, respectively. Overall, the reliability evidence for the final version of the MAANS can be considered moderate.

#### 3.2.7 Predictive validity of the final version of the MAANS

In order to assess the predictive validity of the final version of the MAANS, multiple regression analysis was conducted to examine the relationship between the three factors (subscales) of the final version of the MAANS and the four subscales of the WHO QOL-BREF Malay version (i.e., Physical QOL, Psychosocial QOL, Social QOL, and Environmental QOL). The results of the multiple regression analyses, depicting the relationship between the WHO QOL-BREF Malay version and the final version of the MAANS, are presented in Table 4.

**Table 4:** Multiple regression analysis of the MAANS on the WHO QOL-BREF Malay version

**MAANS subscales**	**Physical** **QOL**		**Psychosocial** **QOL**		**Social** **QOL**		**Environmental** **QOL**	

	**β**	**Sig.**	**B**	**Sig.**	**B**	**Sig.**	**β**	**Sig.**
Perceived Non-Susceptibility	−0.07	0.34	−0.06	0.41	−0.11	0.12	−0.12	0.09
Poor Doctor-Patient Relationship	−0.07	0.31	−0.07	0.36	−0.27	0.00	−0.26	0.00
Unhealthy Lifestyle	−0.10	0.18	−0.11	0.18	−0.22	0.00	−0.22	0.00
*R* ^2^	0.01		0.01		0.15		0.15	

As presented in Table 4, the analyses revealed that none of the subscales of the final version of the MAANS emerged as significant independent predictors of Physical QOL and Psychosocial QOL. However, when Social QOL was regressed on the final version of the MAANS subscales, both Poor Doctor-Patient Relationship (β = −0.27, *p* = 0.00) and Unhealthy Lifestyle (β = −0.22, *p* = 0.00) emerged as the significant predictors of Social QOL. Perceived Non-Susceptibility did not exhibit statistical significance within this model. Furthermore, when regressing Environmental QOL on the subscales of the final version of the MAANS, Poor Doctor-Patient Relationship (β = −0.26, *p* = 0.00) and Unhealthy Lifestyle (β = −0.22, *p* = 0.00) emerged as significant predictors of Environmental QOL. Perceived Non-Susceptibility did not demonstrate statistical significance in this model. Therefore, it was observed that the final version of the MAANS demonstrated only partial predictive validity when regressed against the WHO QOL-BREF Malay version. It is noteworthy that the Perceived Non-Susceptibility subscale did not show a significant prediction for any of the subscales in the WHO QOL-BREF Malay version.

## 4. Discussion

The present study validated a newly developed scale for assessing non-adherence to anti-hypertensive medications among local hypertensive patients as derived from qualitative themes. The final version of the MAANS comprises 14 items. Although the scale demonstrated moderate reliability and partial predictive validity, it offers a multidimensional, patient-centered approach to measuring medication non-adherence in the context of hypertension.

### 4.1 Factor structure of the MAANS

The MAANS has three factors that capture perceived susceptibility, unhealthy lifestyle, and doctor-patient relationship. Firstly, perceived susceptibility refers to an individual’s subjective belief regarding their likelihood of acquiring a disease or facing negative consequences due to specific behaviours [41]. Patients with a strong perception of susceptibility demonstrate higher medication adherence [42–44]. On the other hand, when patients perceive themselves as not susceptible to hypertensive complications, assuming they are unlikely to experience such consequences due to non-adherence, they may consciously or unconsciously neglect taking their anti-hypertensive medications. This lack of perceived susceptibility can diminish their motivation to engage in adherence behaviours. Secondly, unhealthy lifestyle encompasses behaviours and habits characterized by minimal or no physical activity, tobacco use, excessive alcohol consumption, and an unhealthy diet. In Malaysia, recent population health surveys indicate that 25.1% of Malaysians are physically inactive [45], influenced by factors like traffic congestion, air pollution, limited parks or walkways, and inadequate sports or leisure facilities [46]. Furthermore, sedentary activities such as television viewing, video watching, and excessive cell phone usage [47] contribute to an unhealthy lifestyle. These behaviours significantly impact adherence to anti-hypertensive medications [48–51], consequently affecting the overall health of individuals with hypertension. Lastly, the doctor-patient relationship refers to a mutual and consensual partnership between a patient and their physician, where the patient seeks the physician’s assistance, and the physician willingly accepts them as a patient [52]. It is widely recognized that the quality of the doctor-patient relationship has a significant impact on patient satisfaction, medication adherence, and overall health outcomes [53]. A local qualitative study highlighted that hypertensive patients in Malaysia tend to discuss medication-related concerns with volunteers from non-governmental organizations (NGOs) who offer close support, rather than their doctors or pharmacists [54]. Additionally, another study revealed that patients perceived nurses to be friendlier and more attentive compared to other healthcare providers. Consequently, a poor doctor-patient relationship has been associated with lower levels of medication adherence among hypertensive patients, which is a commonly reported finding in previous literature [50, 55, 56].

By comprehending the underlying determinants non-adherence factors, healthcare providers can strategically design interventions that center around sociomedical and preventive medicine, thus enhancing the management of hypertension and preventing hypertension-related complications. For instance, in addressing the factor of perceived susceptibility, healthcare providers can elucidate to patients the significance of adhering to their medication regimen. Meanwhile, tackling the poor doctor-patient relationship factor may entail enhancing communication between doctors and patients, while addressing the unhealthy lifestyle factor could involve educating patients about the importance of adopting healthy habits.

### 4.2 Reliability of the MAANS

The final version of the 14-item MAANS demonstrated moderate reliability, as indicated by Cronbach’s alpha statistics. Specifically, two subscales, namely Perceived Non-Susceptibility and Poor Doctor-Patient Relationship, exhibited moderate reliability. However, the reliability of the Unhealthy Lifestyle subscale was deemed insufficient. It is worth noting that reliability values tend to increase as the test length increases. Nevertheless, in the current scale, increasing the number of items could potentially lead to decreased participation and respondent fatigue due to the length of the scale.

### 4.3 Predictive validity of the MAANS

Considering that medication adherence is often evaluated in relation to quality of life (QOL) [57, 58], the present study aimed to assess the predictive validity of the MAANS by examining its association with QOL measured using the WHO QOL-BREF Malay version. It is worth noting that both the Poor Doctor-Patient Relationship and Unhealthy Lifestyle subscales emerged as significant independent predictors of Social QOL [59, 60]. This can be explained owing to the fact that poor doctor-patient relationship can impede patients’ capacity to effectively manage negative emotions, thus creating a barrier to establishing social connections with others [61]. Meanwhile, an unhealthy lifestyle characterized by an unhealthy diet contributes to weight gain, decreased energy levels, and compromised mental well-being [62, 63], can affect both self-confidence and the motivation to establish new social relationships [64, 65]. Of note, both the Poor Doctor-Patient Relationship and Unhealthy Lifestyle subscales were also found to predict Environmental QOL, which include physical safety, home environment, and engagement in recreational or leisure activities [66]. A poor doctor-patient relationship can hinder thorough assessment by physicians and contribute to patient distrust in diagnosis and treatment, ultimately leading to reduced adherence to medical advice and increased healthcare expenses [67], potentially limiting the patient’s involvement in recreational activities. Adopting unhealthy lifestyle such as a sedentary lifestyle, smoking, excessive alcohol consumption, and an unhealthy diet can contribute to adverse health outcomes, disability, and even mortality [68]. Consequently, individuals or their family members may face considerable income loss, thereby affecting the quality of their home environment and compromising their physical safety and security.

### 4.4 Strengths and limitations

The present study has several notable strengths. Firstly, the development of the MAANS involved a rigorous validation process incorporating both qualitative and quantitative methods, distinguishing it from existing literature, medication adherence scales, and established conceptual frameworks. This unique approach ensures that the MAANS is specifically designed to capture the pertinent aspects of medication adherence in the local context of hypertension. Secondly, the content validity of the MAANS was established through an expert panel. The experts provided both quantitative and qualitative feedback during the review process. They evaluated the content validity indices at the item and subscale levels, and also had the opportunity to provide qualitative feedback on each item. This thorough expert review enhances the confidence in the content validity of the scale. These strengths contribute to the robustness and practicality of the MAANS as a measurement tool for assessing medication adherence in clinical and research settings.

The present study acknowledges several limitations that should be taken into consideration. Firstly, the sample population included in the EFA and CFA may not fully represent the entire Malaysian population. The study was conducted in two health clinics located in an urban setting, which may limit the generalizability of the findings to other regions or populations with different socioeconomic characteristics. Therefore, caution should be exercised when extrapolating the results to the broader population. Secondly, in contrast to scales that include a neutral response as a midpoint, the deliberate exclusion of a neutral option in the MAANS’ four-point Likert-type response format was made to address potential issues associated with neutral responses, which can arise from factors such as ignorance, uncooperativeness, reading difficulty, reluctance to answer, or inapplicability [69, 70]. It is worth noting that the absence of a neutral option may affect participants’ response patterns.

## 5. Conclusion

In summary, 14-item, 3-factor MAANS is a reliable, valid, and multidimensional scale for evaluating non-adherence to anti-hypertensive medications in local clinical settings. It captures the underlying factors of non-adherence and has the potential to be a valuable tool for advancing research and practice in sociomedical and preventive medicine.

## Declarations

### Availability of data and material

Data available on request from authors.

## References

[1] Lee EKP, Poon P, Yip BHK, Bo Y, Zhu MT, Yu CP, . Global Burden, Regional Differences, Trends, and Health Consequences of Medication Nonadherence for Hypertension During 2010 to 2020: A Meta-Analysis Involving 27 Million Patients. J Am Heart Assoc. 2022;11(17):e026582. 36056737 10.1161/JAHA.122.026582 PMC9496433

[2] Turki AK, Sulaiman S. Elevated blood pressure among patients with hypertension in general hospital of Penang, Malaysia: Does poor adherence matter? Int J Pharm Pharm Sci. 2010;2:24–32.

[3] Mohamad Yusoff NH, Joyce Leong WS, Soo MJ, Ching SM. Prevalence and Factors Associated with Medication Non-Compliance among Patients with Hypertension in a Tertiary Hospital: A Cross-Sectional Study in Malaysia. Malays J Med Health Sci. 16(2):36–42.

[4] Alefan Q, Izham M, Mohamed Ibrahim MI, Tariq A, Ayub A. Cost of treating hypertension in Malaysia. Asian J Pharmaceut Clin Res. 2009;2.

[5] S Mehrdad H. Medication Non-adherence: a Major Cause of Resistant Hypertension. Curr Cardiol Rep. 2020;22(11). 10.1007/s11886-020-01400-3 32910342

[6] Mills KT, Stefanescu A, He J. The global epidemiology of hypertension. Nat Rev Nephrol. 2020;16(4):223–37. 32024986 10.1038/s41581-019-0244-2 PMC7998524

[7] Zhou W, Wang W, Fan C, Zhou F, Ling L. Residential elevation and its effects on hypertension incidence among older adults living at low altitudes: a prospective cohort study. Environ Health Prev Med. 2022;27:19. 35527011 10.1265/ehpm.22-00001 PMC9251620

[8] Lee HJ, Jang SI, Park EC. Effect of adherence to antihypertensive medication on stroke incidence in patients with hypertension: a population-based retrospective cohort study. BMJ Open. 2017;7(6):e014486. 10.1136/bmjopen-2016-014486 PMC5734476 28674133

[9] Ge L, Heng BH, Yap CW. Understanding reasons and determinants of medication non-adherence in community-dwelling adults: a cross-sectional study comparing young and older age groups. BMC Health Serv Res. 2023;23(1):905. 37620970 10.1186/s12913-023-09904-8 PMC10464472

[10] Yew SQ, Zulkifley NH. A Review of the Effectiveness of Interventions on Medication Adherence Among Hypertension Patients. 2020.

[11] Svarstad BL, Chewning BA, Sleath BL, Claesson C. The Brief Medication Questionnaire: a tool for screening patient adherence and barriers to adherence. Patient Educ Couns. 1999;37(2):113–24. 14528539 10.1016/s0738-3991(98)00107-4

[12] Morisky DE, Green LW, Levine DM. Concurrent and predictive validity of a self-reported measure of medication adherence. Med Care. 1986;24(1):67–74. 3945130 10.1097/00005650-198601000-00007

[13] Morisky DE, Ang A, Krousel-Wood M, Ward HJ. Predictive validity of a medication adherence measure in an outpatient setting. J Clin Hypertens (Greenwich). 2008;10(5):348–54. 18453793 10.1111/j.1751-7176.2008.07572.x PMC2562622

[14] Wetzels G, Nelemans P, van Wijk B, Broers N, Schouten J, Prins M. Determinants of poor adherence in hypertensive patients: development and validation of the “Maastricht Utrecht Adherence in Hypertension (MUAH)-questionnaire”. Patient Educ Couns. 2006;64(1–3):151–8. 16427764 10.1016/j.pec.2005.12.010

[15] Cabral AC, Castel-Branco M, Caramona M, Fernandez-Llimos F, Figueiredo IV. Developing an adherence in hypertension questionnaire short version: MUAH-16. J Clin Hypertens (Greenwich). 2018;20(1):118–24. 29171719 10.1111/jch.13137 PMC8031096

[16] Ma C, Chen S, You L, Luo Z, Xing C. Development and psychometric evaluation of the Treatment Adherence Questionnaire for Patients with Hypertension. J Adv Nurs. 2012;68(6):1402–13. 21954893 10.1111/j.1365-2648.2011.05835.x

[17] Kim MT, Hill MN, Bone LR, Levine DM. Development and testing of the Hill-Bone Compliance to High Blood Pressure Therapy Scale. Prog Cardiovasc Nurs. 2000;15(3):90–6. 10951950 10.1111/j.1751-7117.2000.tb00211.x

[18] Risser J, Jacobson TA, Kripalani S. Development and psychometric evaluation of the Self-efficacy for Appropriate Medication Use Scale (SEAMS) in low-literacy patients with chronic disease. J Nurs Meas. 2007;15(3):203–19. 18232619 10.1891/106137407783095757

[19] Krousel-Wood M, Joyce C, Holt EW, Levitan EB, Dornelles A, Webber LS, . Development and evaluation of a self-report tool to predict low pharmacy refill adherence in elderly patients with uncontrolled hypertension. Pharmacotherapy. 2013;33(8):798–811. 23649849 10.1002/phar.1275 PMC3729884

[20] Williamson DA, Anton SD, Han H, Champagne CM, Allen R, LeBlanc E, . Adherence is a multi-dimensional construct in the POUNDS LOST trial. J Behav Med. 2010;33(1):35–46. 19856202 10.1007/s10865-009-9230-7 PMC3153914

[21] Cheong AT, Tong SF, Sazlina SG. Validity and reliability of the Malay version of the Hill-Bone compliance to high blood pressure therapy scale for use in primary healthcare settings in Malaysia: A cross-sectional study. Malays Fam Physician. 2015;10(2):36–44. 27099659 PMC4826579

[22] Yew SQ, Mohd Nazan AIN, Tan KA. Study Protocol of a Mixed-Methods Study to Develop and Validate the Malaysian Anti-Hypertensive Agents Non-Adherence Scale in Hypertensive Patients. Malays J Med Health Sci. 2022;18:332–9.

[23] Tabachnick BG, Fidell LS. Using multivariate statistics, 5th ed. Boston, MA: Allyn & Bacon/Pearson Education; 2007 2007. xxvii, 980 p.

[24] Gorsuch RL. Exploratory Factor Analysis. In: Nesselroade JR, Cattell RB, editors. Handbook of Multivariate Experimental Psychology. Perspectives on Individual Differences. Boston, MA: Springer US; 1988. p. 231–58.

[25] Costello AB, Osborne J. Best practices in exploratory factor analysis: four recommendations for getting the most from your analysis. 2005.

[26] Hinkin TR. A review of scale development practices in the study of organizations. J Manage. 1995;21(5):967–88.

[27] Russell DW. In Search of Underlying Dimensions: The Use (and Abuse) of Factor Analysis in Personality and Social Psychology Bulletin. Pers Soc Psychol Bull. 2002;28(12):1629–46.

[28] Cattell RB. The Scree Test For The Number Of Factors. Multivariate Behav Res. 1966;1(2):245–76. 26828106 10.1207/s15327906mbr0102_10

[29] Cattell RB, Jaspers J. A general plasmode (No. 30-10-5-2) for factor analytic exercises and research. Multivariate Behavioral Research Monographs. 1967;67(3):211.

[30] Worthington RL, Whittaker TA. Scale Development Research: A Content Analysis and Recommendations for Best Practices. Couns Psychol. 2006;34(6):806–38.

[31] Zygmont C, Smith MR. Robust factor analysis in the presence of normality violations, missing data, and outliers: Empirical questions and possible solutions. TQMP. 2014;10(1):40–55.

[32] Fonseca-Pedrero E. Structural equation modeling with Mplus: Basic concepts, applications, and programming. Psicothema. 2012;24:343–4.

[33] Ullman JB. Structural equation modeling: reviewing the basics and moving forward. J Pers Assess. 2006;87(1):35–50. 16856785 10.1207/s15327752jpa8701_03

[34] Schumaker R, Lomax R, St C. A beginner’s Guide to Structural Equation Modeling2022 2022/07/18/.

[35] Hu Lt, Bentler PM. Cutoff criteria for fit indexes in covariance structure analysis: Conventional criteria versus new alternatives. Struct Equ Modeling. 1999;6(1):1–55.

[36] Brown TA. Confirmatory factor analysis for applied research, 2nd ed. New York, NY, US: The Guilford Press; 2015 2015. xvii, 462 p.

[37] SPSS Amos - Overview. 2023.

[38] Nunnally JC, Bernstein IH. Psychometric Theory. 3rd edition ed. New York, NY: McGraw-Hill; 1994 1994-01-01. 736 p.

[39] Yew SQ, Tan KA, Mohd Nazan AIN, Abdul Manaf R. Domains of Adherence and Non-Adherence to Anti- Hypertensive Medications in Hypertensive Patients from Kuala Lumpur: A Qualitative Study. Malays J Med Health Sci. 2023;19(5):70–81.

[40] O’connor BP. SPSS and SAS programs for determining the number of components using parallel analysis and Velicer’s MAP test. Behav Res Methods Instrum Comput. 2000;32(3):396–402. 11029811 10.3758/bf03200807

[41] Rosenstock IM. The Health Belief Model and Preventive Health Behavior. Health Educ Monogr. 1974;2(4):354–86. 10.1177/109019817800600406 299611

[42] Kamran A, Sadeghieh Ahari S, Biria M, Malepour A, Heydari H. Determinants of Patient’s Adherence to Hypertension Medications: Application of Health Belief Model Among Rural Patients. Ann Med Health Sci Res. 2014;4(6):922–7. 25506487 10.4103/2141-9248.144914 PMC4250992

[43] Youssef RM, Moubarak II. Patterns and determinants of treatment compliance among hypertensive patients. East Mediterr Health J. 2002;8(4–5):579–92. 15603041

[44] Arindari DR, Suswitha D. Health Belief Model Factors to Medication Adherence among Hypertensive Patients in Punti Kayu Public Health Center Palembang, Indonesia. Jurnal Keperawatan. 2020;11(1):22–7.

[45] Khoo S, Poh BK, Suhaimi SA, Chong KH, Ramirez Varela A. Physical Activity Promotion in Malaysia: Challenges and Opportunities. Front Public Health. 2020;8:536239. 33194945 10.3389/fpubh.2020.536239 PMC7652762

[46] WHO. More physical activity 2023 [Available from: https://www.who.int/teams/health-promotion/physical-activity.

[47] Fennell C, Barkley JE, Lepp A. The relationship between cell phone use, physical activity, and sedentary behavior in adults aged 18–80. Comput Human Behav. 2019;90:53–9.

[48] Ahmad S. Assessment of adherence to antihypertensive treatment among patients attending a health care facility in North India. 2015.

[49] Khanam MA, Lindeboom W, Koehlmoos TLP, Alam DS, Niessen L, Milton AH. Hypertension: adherence to treatment in rural Bangladesh--findings from a population-based study. Glob Health Action. 2014;7:25028. 25361723 10.3402/gha.v7.25028 PMC4212079

[50] Gupta S, Dhamija J, Gupta R. Barriers for adherence to anti-hypertensive medication among rural women in india: Qualitative study. Indian Heart J. 2018;70:S10.

[51] Odusola AO, Hendriks M, Schultsz C, Bolarinwa OA, Akande T, Osibogun A, . Perceptions of inhibitors and facilitators for adhering to hypertension treatment among insured patients in rural Nigeria: a qualitative study. BMC Health Serv Res. 2014;14:624. 25491509 10.1186/s12913-014-0624-z PMC4267751

[52] Honavar SG. Patient–physician relationship – Communication is the key. Indian J Ophthalmol. 2018;66(11):1527–8. 30355854 10.4103/ijo.IJO_1760_18 PMC6213668

[53] Mahmoudian A, Zamani A, Tavakoli N, Farajzadegan Z, Fathollahi-Dehkordi F. Medication adherence in patients with hypertension: Does satisfaction with doctor-patient relationship work? J Res Med Sci. 2017;22:48. 28567067 10.4103/jrms.JRMS_205_16 PMC5426097

[54] Tan CS, Hassali MA, Neoh CF, Saleem F. A qualitative exploration of hypertensive patients’ perception towards quality use of medication and hypertension management at the community level. Pharm Pract (Granada). 2017;15(4):1074. 29317924 10.18549/PharmPract.2017.04.1074 PMC5742001

[55] Saleem F, Hassali MA, Shafie AA, Atif M. Drug Attitude and Adherence: A Qualitative Insight of Patients with Hypertension. J Young Pharm. 2012;4(2):101–7. 22754262 10.4103/0975-1483.96624 PMC3385213

[56] de Guzman AB, Guevara KIJ, Guiang FJB, Gutierrez ALI, Habaluyas AS, Hizon MAP, . Developing a Model of Medication Adherence among Filipino Elderly. Educ Gerontol. 2013;39(5):298–313.

[57] Ha J, Lee C, Park W, Suh J, Choi E, Jeon D. . Medication Adherence and Quality of Life of Uncontrolled Hypertension Patients in Korea. Value in Health. 2017;20:A621.

[58] Khayyat SM, Mohamed MMA, Khayyat SMS, Hyat Alhazmi RS, Korani MF, Allugmani EB, . Association between medication adherence and quality of life of patients with diabetes and hypertension attending primary care clinics: a cross-sectional survey. Qual Life Res. 2019;28(4):1053–61. 30470970 10.1007/s11136-018-2060-8

[59] Farin E, Meder M. Personality and the physician-patient relationship as predictors of quality of life of cardiac patients after rehabilitation. Health Qual Life Outcomes. 2010;8:100. 20840774 10.1186/1477-7525-8-100 PMC2949817

[60] Zhang B, Nilsson ME, Prigerson HG. Factors Important to Patients’ Quality-of-Life at the End-of-Life. Arch Intern Med. 2012;172(15):1133–42. 22777380 10.1001/archinternmed.2012.2364 PMC3806298

[61] Bonsu AB, Aziato L, Clegg-Lamptey JNA. Living with Advanced Breast Cancer among Ghanaian Women: Emotional and Psychosocial Experiences. Int J Palliative Care. 2014;2014:e403473.

[62] Barlow P, Reeves A, McKee M, Galea G, Stuckler D. Unhealthy diets, obesity and time discounting: a systematic literature review and network analysis. Obes Rev. 2016;17(9):810–9. 27256685 10.1111/obr.12431 PMC4988386

[63] Cabello M, Miret M, Caballero FF, Chatterji S, Naidoo N, Kowal P, . The role of unhealthy lifestyles in the incidence and persistence of depression: a longitudinal general population study in four emerging countries. Global Health. 2017;13:18. 28320427 10.1186/s12992-017-0237-5 PMC5358047

[64] Wahl DR, Villinger K, König LM, Ziesemer K, Schupp HT, Renner B. Healthy food choices are happy food choices: Evidence from a real life sample using smartphone based assessments. Sci Rep. 2017;7(1):17069. 29213109 10.1038/s41598-017-17262-9 PMC5719018

[65] Grimani A, Aboagye E, Kwak L. The effectiveness of workplace nutrition and physical activity interventions in improving productivity, work performance and workability: a systematic review. BMC Public Health. 2019;19(1):1676. 31830955 10.1186/s12889-019-8033-1 PMC6909496

[66] Skevington SM, Lotfy M, O’Connell KA, Group W. The World Health Organization’s WHOQOL-BREF quality of life assessment: psychometric properties and results of the international field trial. A report from the WHOQOL group. Qual Life Res. 2004;13(2):299–310. 15085902 10.1023/B:QURE.0000018486.91360.00

[67] Iuga AO, McGuire MJ. Adherence and health care costs. Risk Manag Healthc Policy. 2014;7:35–44. 24591853 10.2147/RMHP.S19801 PMC3934668

[68] Farhud DD. Impact of Lifestyle on Health. Iran J Public Health. 2015;44(11):1442–4. 26744700 PMC4703222

[69] DeCastellarnau A. A classification of response scale characteristics that affect data quality: a literature review. Qual Quant. 2018;52(4):1523–59. 29937582 10.1007/s11135-017-0533-4 PMC5993837

[70] Frary R. Hints for Designing Effective Questionnaires. Pract Assess Res Eval. 2019;5(1).

